# Physicochemical Properties of Epoxy Resin-Based and Bioceramic-Based Root Canal Sealers

**DOI:** 10.1155/2017/2582849

**Published:** 2017-01-22

**Authors:** Ju Kyung Lee, Sang Won Kwak, Jung-Hong Ha, WooCheol Lee, Hyeon-Cheol Kim

**Affiliations:** ^1^Department of Conservative Dentistry, School of Dentistry, Pusan National University, Dental Research Institute, Yangsan, Republic of Korea; ^2^Department of Conservative Dentistry, School of Dentistry, Kyungpook National University, Daegu, Republic of Korea; ^3^Department of Conservative Dentistry, Seoul National University School of Dentistry, Seoul, Republic of Korea

## Abstract

Three bioceramic sealers (EndoSequence BC sealer, EndoSeal MTA, and MTA Fillapex) and three epoxy resin-based sealers (AH-Plus, AD Seal, and Radic-Sealer) were tested to evaluate the physicochemical properties: flow, final setting time, radiopacity, dimensional stability, and pH change. The one-way ANOVA and Tukey's post hoc test were used to analyze the data (*P* = 0.05). The MTA Fillapex sealer had a highest flow and the BC Sealer presented a flow significantly lower than the others (*P* < 0.05). The BC Sealer and MTA Fillapex samples were not set in humid incubator condition even after one month. EndoSeal MTA had the longest setting time among the measurable materials and Radic-Sealer and AD Seal showed shorter setting time than the AH-Plus (*P* < 0.05). AH-Plus and EndoSeal MTA showed statistically higher values and MTA Fillapex showed statistically lower radiopacity (*P* < 0.05). BC Sealer showed the highest alkaline pH in all evaluation periods. Set samples of 3 epoxy resin-based sealers and EndoSeal MTA presented a significant increase of pH over experimental time for 4 weeks. In conclusion, the bioceramic sealer and epoxy resin-based sealers showed clinical acceptable physicochemical properties, but BC Sealer and MTA Fillapex were not set completely.

## 1. Introduction

Endodontic sealers are used to attain a fluid-proof seal throughout root canal system [1]. An ideal root canal sealer should offer an excellent seal when set, dimensional stability, a sufficient setting time to ensure working time, insolubility against tissue fluids, proper adhesion with canal walls, and biocompatibility [2, 3].

The commercially available sealers are categorized according to chemical components: zinc-oxide eugenol, calcium hydroxide containing, resin-based, glass-ionomer-based, silicone-based, and bioceramic-based sealers [4–6]. Epoxy resin-based sealers were introduced in endodontics by Schroeder [7], and current modifications of the original formula are widely used for root canal filling procedures [8, 9]. Recently, bioceramic-based materials such as EndoSequence BC Sealer (Brasseler USA, Savannah, GA), EndoSeal MTA (MARUCHI, Wonju, Korea), and MTA Fillapex (Angelus Soluções Odontológicas, Londrina, PR, Brazil) have received considerable attention because of their favorable physicobiological properties [10, 11]. Among them, EndoSequence BC Sealer and EndoSeal MTA are supplied in a premixed injectable paste and thus give clinicians easy manipulation. These currently introduced calcium silicate based sealers still have few reports about their chemical and physical properties [3, 10, 12].

This study aimed to evaluate the physicochemical properties of 3 epoxy resin-based sealers and 3 bioceramic-based sealers according to the international standards such as ISO 6876/2012 standards [13] and ANSI/ADA's specifications number 57 [14] (Table 1).

## 2. Materials and Methods

**Figure 1:**
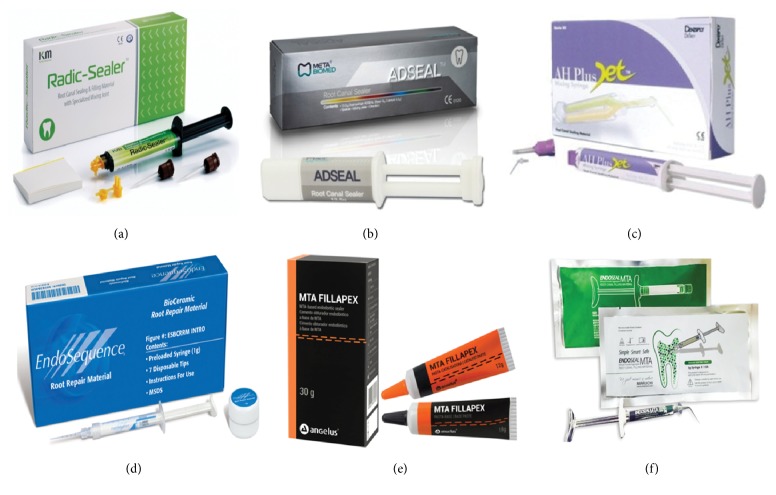
Six root canal sealers tested in the present study: (a) Radic-Sealer, (b) AD Seal, (c) AH-Plus, (d) EndoSequence BC Sealer, (e) MTA Fillapex, and (f) EndoSeal MTA.

Three epoxy resin-based root canal sealers of AH-Plus, AD Seal, and Radic-Sealer and 3 bioceramic-based sealers of EndoSequence BC Sealer, EndoSeal MTA, and MTA Fillapex were used as the experimental materials (Figure 1, Table 2). AH-Plus (Dentsply DeTrey, Konstanz, Germany) is the most popular hydrophobic epoxy resin-based sealer that has been used as the gold standard material [3]. AD Seal (Meta Biomed, Cheongju, Korea) and Radic-Sealer (KM, Seoul, Korea) are the epoxy resin-based sealers with few reports in literature [15, 16].

The physicochemical properties including flow, final setting time, radiopacity, dimensional stability, and pH change were examined according to modified ISO 6876/2012 standards [13] and ANSI/ADA's specifications number 57 [14]. All sealers were mixed and manipulated depending on the manufacturers' instructions.

### 2.1. Flow

A volume of 0.05 mL mixed sealer was dropped on a glass plate. At 3 minutes after the onset of mixing, a second glass plate was placed on the sealer and a 100 g weight was added to make a total mass of 120 g. The 120 g weight was unloaded after 10 minutes from the start of mixing. The minimum and maximum diameters of the sealer disc were measured by a digital caliper (Mitutoyo Corp, Tokyo, Japan) with a resolution of 0.01 mm. If the disks were not uniformly circular, the test was repeated. Ten tests were taken for each sealer.

### 2.2. Final Setting Time

Stainless steel ring molds (inner diameter 10 mm, height 2 mm) were placed on a glass plate, and then the sealer materials were mixed and packed into the molds. The whole assembly was then stored in an incubator (37°C, >95% relative humidity) for at least 1 hour. To measure the setting time, the needle of a custom-made Vicat apparatus was adjusted vertically onto the surface of the sealer. The setting time was determined as the time when the indenter needle failed to create an indentation. The measurement interval was adjusted from 1 hour at the beginning to 5 min in accordance with the setting process. The time from the onset of mixing to the sealer setting was taken as the setting time. Ten measurements were made for each sealer.

### 2.3. Radiopacity

Ten cylindrical samples were fabricated from each sealer by placing the handled sealers into metallic rings with 8 mm internal diameter and 1 mm thickness. Then the filled rings were stored at 37°C until sealers were completely set. The samples were radiographed on a digital X-ray sensor (Schick Technology Inc., Long Island City, NY) with an aluminum step-wedge graduated from 1 mm to 10 mm (in 1 mm increment), which was used with exposures set at 60 kV, 2 mA, 0.08 seconds, and a focus-film distance of 10 cm. The aluminum wedge equivalent thickness (mm Al) of each sealer was analyzed by using Photoshop (Adobe photo shop 7.0; Adobe systems Incorporated, San Jose, CA).

### 2.4. Dimensional Stability

Dimensional stability was measured for the settable sealers of 3 epoxy resin-based sealers and EndoSeal MTA. Cylindrical Teflon molds (inner diameter 6 mm, height 12 mm) were filled with mixed sealer and backed by a glass plate on each side. The whole assembly was transferred to an incubator and kept for at least 3 times the measured setting time. After deciding the complete setting, the ends of the molds containing the specimens were ground by using 600-grit sandpaper with water supply. Then the specimens were removed from the mold and measured for length (*L*_0_) using a digital caliper with a resolution of 0.01 mm. The specimens were stored in distilled water and kept in an incubator throughout the study period (6, 24, and 72 hours and 7, 14, and 30 days). After being immersed in water for the assigned periods, the dimensions of 4 tested sealers were compared to their initial dimension. The samples were then blotted dry with tissue paper and measured again for length (*L*_1_). The test was implemented ten times for each sealer, and the change in length was recorded as the dimensional change (*D*) using the following formula: *D*(%) = (*L*_1_ − *L*_0_)/*L*_0_ × 100.

### 2.5. pH Change

The sealer samples mixed immediately after manipulation were denoted as fresh samples, and the samples stored in the incubator until setting were denoted as set samples. Teflon molds (inner diameter 5 mm, thickness 1 mm) were used to shape the set samples. Both the set sample and fresh sample were dropped in distilled water in a polypropylene conical tube and then stored at 37°C throughout the study period. After predetermined periods (3, 30, and 60 minutes and 2, 12, and 24 hours for fresh samples and 12 hours, 3 days, 7 days, 2 weeks, and 4 weeks for set samples), the pH of the solution was measured by using a digital pH meter (STARTER 2100 Bench pH Meter; Ohaus). Ten measurements were made for each sealer and condition.

### 2.6. Statistical Analysis

The one-way ANOVA test and Tukey's post hoc test were used to compare the physicochemical property results by using SPSS software 10.0 (SPSS Inc., Chicago, IL). The significance level was set at *P* < 0.05.

## 3. Results

The physical properties of the sealers are summarized in Table 3.

**Figure 2:**
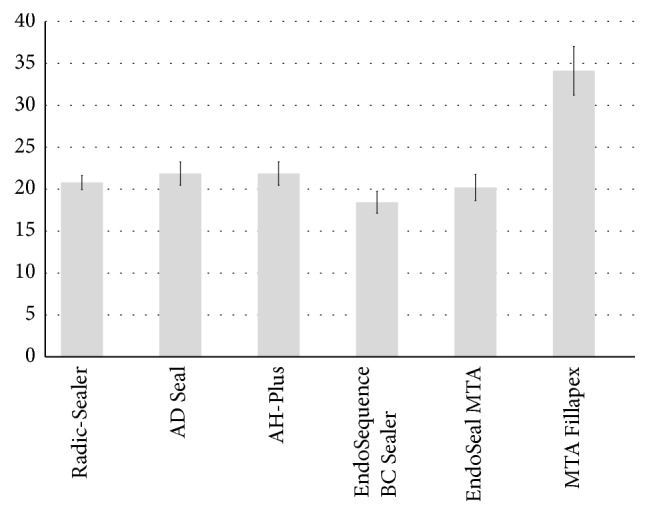
Flow values from each sealer evaluated (in mm). ^a, b, c^Different letters present significant difference between groups (*P* < 0.05).

All the tested sealers except BC Sealer showed the flow greater than 20 mm, which is in agreement with the ISO standards [13]. MTA Fillapex had a highest flow and BC Sealer presented a significantly lower flow than the other sealers (*P* < 0.05) (Figure 2).

BC Sealer and MTA Fillapex were not set in humid incubator condition even after one month. EndoSeal MTA had the longest setting time (mean: 1223 min) among the measurable materials and Radic-Sealer and AD Seal showed shorter setting time than the AH-Plus (*P* < 0.05).

**Figure 3:**
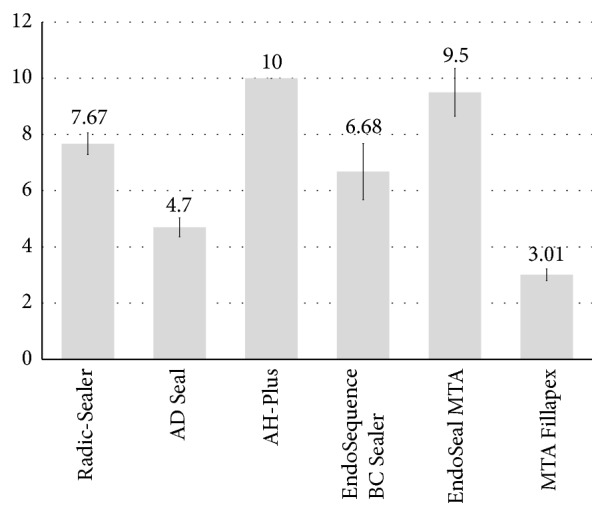
Radiopacity values from each sealer evaluated (in mm Al). ^a, b, c, d, e^Different letters present significant difference between groups (*P* < 0.05).

For the radiopacity test, AH-Plus and EndoSeal MTA showed statistically higher values and MTA Fillapex showed statistically lower values in comparison to the other evaluated sealers (*P* < 0.05) (Figure 3). All the tested sealers showed radiopacity values complying with the ISO standards [13].

**Figure 4:**
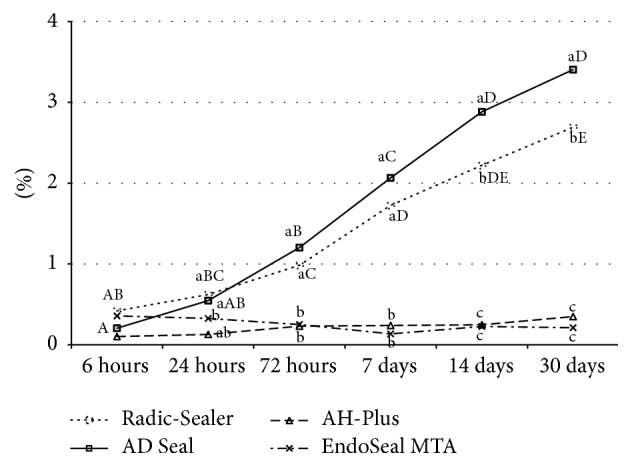
Dimensional stability (%) of the test sealers at various time periods. Different letters present significant differences between groups (a, b, and c) at the same period and between the time periods (A, B, C, D, and E) in the same material (*P* < 0.05).

Dimensional stability was measured for the settable sealers of 3 epoxy resin-based sealers and EndoSeal MTA. After being immersed in water for 30 days, 4 tested sealers expanded compared to their initial dimension. At 30 days, AD Seal had a significantly greater expansion than the others (*P* < 0.05) (Table 4, Figure 4).

**Figure 5:**
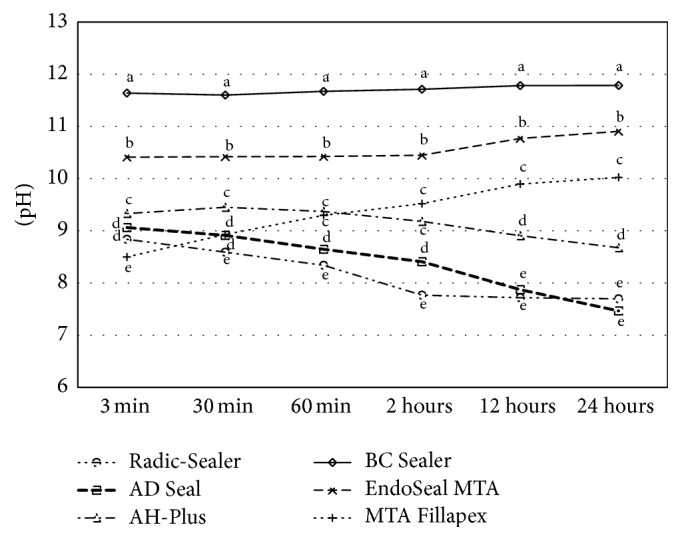
pH change of freshly mixed samples during 24 hours. ^a, b, c, d, e^Different letters present significant difference between sealers at the tested period (*P* < 0.05).

**Figure 6:**
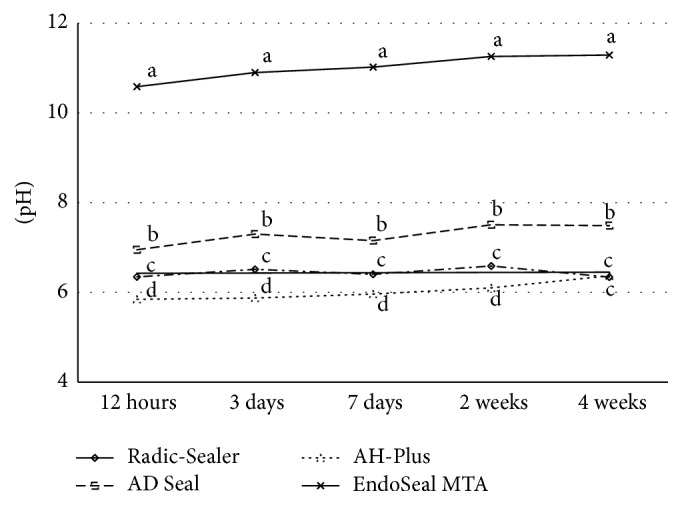
pH change of set samples during 4 weeks. ^a, b, c, d^Different letters present significant difference between four sealers at the same test time periods (*P* < 0.05).

Fresh samples of the tested sealers showed significant differences of pH change among themselves at all evaluation time points and BC Sealer showed the highest alkaline pH in all evaluation periods (Table 5, Figure 5). Set samples of 3 epoxy resin-based sealers and EndoSeal MTA presented a significant increase of pH over experimental time for 4 weeks. The pH of EndoSeal MTA was significantly higher than that of 3 epoxy resin-based root canal sealers at all experimental time points. Radic-Sealer and AH-Plus showed mild acidity around pH 6 and AD Seal presented neutral pH at 4 weeks (*P* < 0.05) (Table 6, Figure 6).

## 4. Discussion

Among the clinically available root canal sealers, epoxy resin-based sealers are widely used for root canal filling due to their resorption resistance and dimensional stability [4–6]. Most recently introduced bioceramic-based materials have attractive physical, chemical, mechanical, and biological properties [10, 11, 17]. Therefore, the representative 3 epoxy resin-based sealers and 3 bioceramic-based sealers were compared for physical and chemical properties, in this study.

The flow of endodontic sealers may have an effect on obturation of accessory canals and microspaces between master and accessory cones [3]. Various factors such as composition, shear rate, particle size, temperature, and time from mixing are related to the flowability of sealers [3]. MTA Fillapex sealer had the highest flow and BC Sealer presented the lowest flow in this study. The flow value of MTA Fillapex was similar to the value obtained by Silva et al. [18]. A high resin/MTA ratio may be one of the reasons why a high flow rate occurs [19].

Setting time is also important to provide adequate working time and proper consistency enough to fill the root canal system completely [20]. Setting times of evaluated sealers in this study were different from that given by the manufacture. Only AH-Plus was in agreement with the ISO standards and it showed a significant higher mean setting time value, almost 8 times greater than the other epoxy resin-based root canal sealers. AH-Plus is comprised of base and catalyst in which a slow polymerization reaction of epoxy resin amines with a high molecular weight including bisphenol A and bisphenol F occurs [21]. This chemical composition could explain significantly higher setting time of it. On the other hand, Radic-Sealer and AD Seal are the kind of resin composites containing a catalyst component that accelerates the process [22]. In the meanwhile, BC Sealer and MTA Fillapex were not set in humid incubator condition, and this result was different from several reports that final setting of these materials occurred [4–6, 10, 23, 24]. Depending on Loushine et al. [10], water is essential for this sealer to reach its final set because the inorganic and radiopacifier components of the sealer are premixed with water-free liquid-thickening carriers, and the manufacturer suggests that there is a prolonged setting time in overly dry canals. However, the authors concluded that overly wet canals may affect the setting time and, in particular, adversely affect the microhardness of the sealer after setting [10]. They also pointed out that a more porous matrix would be present when the sealer sets in the wet canals, which, in turn, may result in increased leaching of tissue-irritating substances from the sealer [10]. The delayed setting time of sealers may also affect biocompatibility and the sealers may have the potential to release cytotoxic byproducts before the final setting [10]. Silva et al. [18] reported that MTA Fillapex showed severe cytotoxicity when cells were exposed to the fresh sealer and the toxicity was not decreased over the tested time periods. These findings are in agreement with other previous studies [25, 26] that showed strongly affected cell viability with MTA Fillapex.

Radiopacity is an essential property of endodontic sealing materials. Among other physical, chemical, and biological properties, the ideal root canal sealing material should have a certain level of radiopacity [27]. Sufficient radiopacity allows clinicians to make a clear distinction between the materials and the surrounding anatomic structures and to evaluate the quality of the root fillings [28]. International standards require a minimal radiopacity equivalent to 3.00 mmAl [29]. In the present study, AH-Plus and EndoSeal MTA showed statistically higher radiopacity values (*P* < 0.05), but all the tested sealers exhibited values complying with the international standards. Vitti et al. [19] suggested that the differences between radiopacities of root canal sealers probably were caused by the presence of different radiopacifying agents in each material. According to Duarte et al. [30], radiopacity of AH-Plus is provided by zirconium oxide and calcium tungstate and suggested that its radiopacity could vary in different published studies because of the deposition of radiopacifying agents at the lower end of the tube, whereas the upper portion can present a lower quantity of its substance [19].

In this study, 4 tested sealers expanded compared to initial dimension and AD Seal had a significant increase of height (i.e., expansion) than the others. This increase of mass and height presented by 3 epoxy resin-based sealers probably occurred as a result of the water absorption and a high expansion of resin-based sealers, which was also verified by Versiani et al. [31]. AH-Plus maintained the most constant mass, presenting mass change rate within −0.5% (minus value means water sorption) for 30 days in this study. Dimensional change values ranging from 0.62% to 1.28% for AH-Plus obtained in previous investigations were also explained by water sorption after polymerization [31, 32]. It has been demonstrated that polymerized materials from mixtures of hydrophilic monomers had high water sorption [33]. And the dimensional change of EndoSeal MTA was not significant with the minimal change of specimens' height in this study. However, all the tested materials showed bigger expansion rate than the favorable rate suggested by the international standards (Table 1). Therefore, it is highly recommended to study the potential risk of inducing the vertical root fractures by the sealer expansion.

An alkaline pH may contribute to their osteogenic potential, biocompatibility, and antibacterial ability [3, 34–37]. It has been reported that an alkaline pH of root canal sealers could neutralize the lactic acid from osteoclasts and prevent dissolution of mineralized components of teeth. Therefore, root canal sealers can contribute to hard tissue formation by activating alkaline phosphatase [38]. In this study, the pH value of 3 freshly prepared bioceramic-based root canal sealers remained significantly higher than that of 3 epoxy resin-based sealers for 24 hours, with the highest alkaline pH measured from BC Sealer for the entire period of evaluation. Considering the setting time required, BC Sealer with prolonged high pH (up to 12) before its setting may cause damage to the periapical tissue via the loss of cell viability and membrane integrity, similar to cellular responses observed in chemical burns. Such complications thus need to be carefully considered, along with bactericidal effect of the sealers. In case of set samples, the pH of EndoSeal MTA was significantly higher than that of 3 epoxy resin-based root canal sealers at all experimental time points (*P* < 0.05).

## 5. Conclusion

Based on the present results, the tested epoxy resin-based sealers as well as the bioceramic-based sealers except the BC Sealer and MTA Fillapex are showed to fulfill the required chemical and physical properties as ideal root canal sealers. The EndoSequence BC Sealer and MTA Fillapex should be improved to be set finally within clinically acceptable time limit. Clinical trial tests and long term follow-up studies using various types of the sealers would be highly valuable to evaluate the sealers' clinical performances.

## Figures and Tables

**Table 1:** ISO 6876/2012 and ANSI/ADA specification number 57 standards.

	ISO standards	ANSI/ADA standards
Setting time	When ≤30 min, ≤110% stated by the manufacture	Within 10% of setting time stated by the manufacturers
	When >30 min, <72 hours, within the range (min)	
Flow	≥20 mm	≥25 mm
Solubility	≤3% for 24 hours	≤3% for 24 hours
Dimensional change	Shrinkage (contraction) ≤1% for 30 days	
	Expansion ≤ 0.1% for 30 days	
Radiopacity	≥3 mm aluminum thickness	

**Table 2:** Chemical compositions of the root canal sealers investigated in the present study.

Sealer		Components	
*Epoxy resin-based sealer*	*Radic-Sealer*	*Base*	*Catalyst*
		Poly epoxy resin Zirconium oxide	TEA (triethanolamine) Zirconium oxide Calcium oxide
	*AD Seal*	*Base*	*Catalyst*
		<20% epoxy resin NS calcium phosphate NS zirconium dioxide NS calcium oxide NS ethylene glycol salicylate	2.5%–10% N, n-dibenzyl-5-oxanonandiamin-1,9 2.5%–10% amantadine
	*AH-Plus*	*Paste A*	*Paste B*
		25%–50% bisphenol A 10%–25% zirconium dioxide NS calcium tungstate NS iron oxide	2.5%–10% N, n-dibenzyl-5-oxanonandiamin-1,9 2.5%–10% amantadine
*Bioceramic-based sealer*	*EndoSequence BC Sealer*	Zirconium oxide, calcium silicates, calcium phosphate monobasic, calcium hydroxide, filler, thickening agents	
	*EndoSeal MTA*	Calcium silicates, calcium aluminates, calcium aluminoferrite, calcium sulfates, radiopacifier, thickening agents	
	*MTA Fillapex*	Salicylate resin, diluting resin, natural resin, bismuth trioxide, nanoparticulated silica, MTA	

**Table 3:** The flow (mm), setting time (min), and radiopacity (mmAl thickness) of tested sealers (mean ± standard deviation).

	Radic-Sealer	AD Seal	AH-Plus	EndoSequence BC Sealer	EndoSeal MTA	MTA Fillapex
Flow (mm)	20.80 ± 0.84^b^	21.87 ± 1.40^b^	21.87 ± 1.40^b^	18.45 ± 1.31^c^	20.21 ± 1.57^b^	34.13 ± 2.91^a^
Setting time (min)	114.1 ± 2.8^c^	115.7 ± 2.8^c^	959.6 ± 79.0^b^	—	1223.4 ± 156.3^a^	—
Radiopacity (mmAl)	7.67 ± 0.38^b^	4.70 ± 0.33^d^	10.00^a*∗*^	6.68 ± 0.99^c^	9.50 ± 0.84^a^	3.01 ± 0.20^e^

^a, b, c, d, e^Different letters in each line indicate significant difference (*P* < 0.05).

^*∗*^All of the AH plus samples had 10 mmAl thickness or the higher values.

**Table 4:** The dimensional stability (%) of the tested sealers at various time periods (mean ± standard deviation).

Ratio (%)	6 hours	24 hours	72 hours	7 days	14 days	30 days
Radic-Sealer	0.42 ± 0.33^AB^	0.62 ± 0.35^aBC^	0.98 ± 0.28^aC^	1.73 ± 0.39^aD^	2.22 ± 0.28^bDE^	2.69 ± 0.32^bE^
AD Seal	0.21 ± 0.27^A^	0.55 ± 0.32^aAB^	1.20 ± 0.39^aB^	2.07 ± 0.56^aC^	2.88 ± 0.54^aD^	3.41 ± 0.76^aD^
AH-Plus	0.10 ± 0.64	0.13 ± 0.65^b^	0.23 ± 0.55^b^	0.24 ± 0.64^b^	0.25 ± 0.54^c^	0.35 ± 0.51^c^
EndoSeal MTA	0.36 ± 0.32	0.33 ± 0.28^ab^	0.25 ± 0.34^b^	0.14 ± 0.33^b^	0.23 ± 0.29^c^	0.21 ± 0.31^c^

^a, b, c^Different letters in each column indicate significant difference between groups at the same period (*P* < 0.05).

^A, B, C, D, E^Different capital letters indicate significant difference during the time periods in the same material (*P* < 0.05).

**Table 5:** pH change of freshly mixed samples during 24 hours.

	3 min	30 min	60 min	2 hours	12 hours	24 hours
Radic-Sealer	8.84 ± 0.25^d^	8.59 ± 0.27^e^	8.34 ± 0.13^e^	7.77 ± 0.34^e^	7.72 ± 0.17^e^	7.70 ± 0.26^e^
AD Seal	9.06 ± 0.47^d^	8.91 ± 0.55^d^	8.65 ± 0.67^d^	8.41 ± 0.92^d^	7.87 ± 0.68^e^	7.46 ± 0.77^e^
AH-Plus	9.33 ± 0.28^c^	9.45 ± 0.26^c^	9.37 ± 0.23^c^	9.18 ± 0.37^c^	8.91 ± 0.46^d^	8.68 ± 0.60^d^
BC Sealer	11.64 ± 0.03^a^	11.60 ± 0.02^a^	11.67 ± 0.03^a^	11.7 ± 0.03^a^	11.78 ± 0.03^a^	11.78 ± 0.03^a^
EndoSeal MTA	10.41 ± 0.05^b^	10.42 ± 0.06^b^	10.42 ± 0.07^b^	10.45 ± 0.07^b^	10.77 ± 0.06^b^	10.90 ± 0.05^b^
MTA Fillapex	8.50 ± 0.26^e^	8.93 ± 0.13^d^	9.30 ± 0.15^c^	9.52 ± 0.18^c^	9.90 ± 0.11^c^	10.02 ± 0.23^c^

^a, b, c, d, e^Different letters in each column indicate significant difference between sealer groups at the tested period (*P* < 0.05).

**Table 6:** pH change of set samples during 4 weeks.

	Initial	12 hours	3 days	7 days	2 weeks	4 weeks
Radic-Sealer	5.79 ± 0.06	6.35 ± 0.09^c^	6.51 ± 0.12^c^	6.40 ± 0.13^c^	6.59 ± 0.51^d^	6.34 ± 0.39^c^
AD Seal	5.84 ± 0.57	6.95 ± 0.83^b^	7.30 ± 0.75^b^	7.15 ± 0.74^b^	7.51 ± 0.86^c^	7.49 ± 0.74^b^
AH-Plus	5.84 ± 0.04	5.85 ± 0.35^d^	5.87 ± 0.47^d^	5.96 ± 0.44^d^	6.10 ± 0.94^b^	6.40 ± 0.47^c^
EndoSeal MTA	5.76 ± 0.11	10.58 ± 0.06^a^	10.90 ± 0.05^a^	11.02 ± 0.04^a^	11.26 ± 0.04^a^	11.29 ± 0.07^a^

^a, b, c, d^Different letters in each column indicate significant difference between sealers at the tested period (*P* < 0.05).
